# Liver regeneration accelerates hepatitis B virus‐related tumorigenesis of hepatocellular carcinoma

**DOI:** 10.1002/1878-0261.12318

**Published:** 2018-05-29

**Authors:** Chiao‐Fang Teng, Hong‐Yi Chang, Hung‐Wen Tsai, Wen‐Chuan Hsieh, Yu‐Hao Kuo, Ih‐Jen Su, Yih‐Jyh Lin

**Affiliations:** ^1^ Graduate Institute of Biomedical Sciences China Medical University Taichung Taiwan; ^2^ Organ Transplantation Center China Medical University Hospital Taichung Taiwan; ^3^ Department of Biotechnology Southern Taiwan University of Science and Technology Tainan Taiwan; ^4^ Department of Pathology National Cheng Kung University Hospital Tainan Taiwan; ^5^ Institute of Clinical Medicine College of Medicine National Cheng Kung University Tainan Taiwan; ^6^ National Institute of Infectious Diseases and Vaccinology National Health Research Institutes Tainan Taiwan; ^7^ Division of General and Transplant Surgery Department of Surgery National Cheng Kung University Hospital Tainan Taiwan; ^8^ Department of Surgery College of Medicine National Cheng Kung University Tainan Taiwan; ^9^ Liver Cancer Collaborative Oncology Group National Cheng Kung University Hospital Tainan Taiwan

**Keywords:** hepatitis B virus, hepatocellular carcinoma, liver regeneration, partial hepatectomy

## Abstract

Although partial hepatectomy (PH) to remove tumors provides a potential cure of hepatocellular carcinoma (HCC), long‐term survival of hepatitis B virus (HBV)‐related HCC patients after PH remains a big challenge. Early recurrence within 2 years post‐PH is associated with the dissemination of primary HCC. However, late recurrence after 2 years post‐PH is supposed due to the *de novo* or a secondary tumor. Since PH initiates liver regeneration (LR), we hypothesize that LR may accelerate tumorigenesis through activation of pre‐existing precancerous lesions in the remaining liver. In this study, we explored the potential role of several LR‐related factors in the *de novo* recurrence in a HBV X protein (HBx) transgenic mouse model receiving PH to mimic human HCC development. Following PH, we observed that tumor development was significantly accelerated from 16.9 to 10.4 months in HBx transgenic mice. The expression of suppressor of cytokine signaling (SOCS) family proteins was remarkably suppressed in livers of HBx transgenic relative to non‐transgenic mice from early to late stages after PH as compared with non‐PH mice. The expression of transforming growth factor‐β (TGF‐β)/Smad pathway, hepatocyte growth factor (HGF), Myc, signal transducer and activator of transcription 3 (STAT3), and β‐Catenin also showed a significant difference between livers of HBx transgenic and non‐transgenic mice at variable time points after PH in comparison with non‐PH mice. Taken together, our results provide an explanation for the high *de novo* recurrence of HBV‐related HCC after PH, probably through induction of the sequential changes of LR‐related SOCS family proteins, growth factors, and transcription factors, which may promote growth on the precancerous remnant liver.

**Abbreviations**
ALT: alanine aminotransferase
HBV: hepatitis B virus
HBx: hepatitis B X protein
HCC: hepatocellular carcinoma
HCV: hepatitis C virus
HGF: hepatocyte growth factor
LR: liver regeneration
PGC1‐α: peroxisome proliferator‐activated receptor‐γ coactivator‐1α
PH: partial hepatectomy
SD: standard deviation
SOCS: suppressor of cytokine signaling
STAT3: signal transducer and activator of transcription 3
TGF‐β: transforming growth factor‐β
VEGF‐A: vascular endothelial growth factor‐A

## 1 Introduction

Hepatocellular carcinoma (HCC) ranks top in the 10 leading causes of cancer‐related deaths worldwide and develops primarily in patients with chronic hepatitis B or C virus (HBV or HCV) infection (El‐Serag, 2012). Although partial hepatectomy (PH) provides a potentially curative procedure for the treatment of HCC (Kishi *et al*., 2011), the recurrence rate 5 years after surgery is as high as 70%, contributing to poor patient survival (Grazi *et al*., 2001; Nakashima *et al*., 2003; Tang *et al*., 2004). It is therefore critical and urgently needed to explore the underlying mechanism of high recurrence of HCC after PH.

HCC recurrence after PH can be classified into early and late recurrence according to the timing of recurrence by 2‐year cut‐off (Bruix *et al*., 2005; Hoshida *et al*., 2010). Early recurrence is usually associated with the dissemination of ‘primary’ tumor or intrahepatic invasion, whereas late recurrence occurs mainly due to multicentric carcinogenesis or ‘*de novo*’ recurrence from the precursor dysplastic lesions in the remaining liver after PH. Although the molecular signatures associated with early HCC recurrence have been characterized (Iizuka *et al*., 2003; Kim *et al*., 2015), few reports concern the predictors or mechanisms for the *de novo* HCC recurrence.

Several mechanisms have been proposed to explain the *de novo* recurrence of HCC after PH, e.g. cancer stem cells, epithelial–mesenchymal transition, and the dysregulation of liver regeneration (LR)‐related proteins (Michalopoulos, 2010). There are a series of scenarios of LR‐related molecular events operating after PH (Yin *et al*., 2013). Among them, the suppressor of cytokine signaling (SOCS) family proteins, including SOCS1–7, which function as negative regulators or tumor suppressors to suppress cytokine‐mediated cell growth, play a key role in the termination of LR at the late phase after PH (Kurinna and Barton, 2011). In addition, several growth and transcription factors, such as transforming growth factor‐β (TGF‐β)/Smad pathway, vascular endothelial growth factor‐A (VEGF‐A), hepatocyte growth factor (HGF), Myc, peroxisome proliferator‐activated receptor‐γ coactivator‐1α (PGC1‐α), signal transducer and activator of transcription 3 (STAT3), and β‐Catenin have also been reported to be related to LR after PH (Bockhorn *et al*., 2007; Fausto *et al*., 2006; Kurinna and Barton, 2011; Moh *et al*., 2007; Monga *et al*., 2001; Sanders *et al*., 2012; Zhong *et al*., 2010). However, the role of these LR‐related factors in HBV‐related HCC recurrence after PH has not yet been well elucidated.

Recently, the ground glass hepatocytes, which harbor two HBV oncoproteins, the pre‐S mutant surface antigen and the hepatitis B X protein (HBx), have been proposed as the precursor lesions of HCC (Wu *et al*., 2014; Yen *et al*., 2016). HBx is an etiologic factor of HBV‐related hepatocarcinogenesis through its multiple functions in regulation of cell cycle, cell proliferation, lipid metabolism, signal transduction, and gene transcription (Arbuthnot *et al*., 2000; Chisari *et al*., 1989; Teng *et al*., 2016). Overexpression of HBx can cause tumorigenicity in an HBx transgenic mouse model (Newell *et al*., 2008). Moreover, HBx expression has been reported to contribute to HCC development by affecting LR (Hodgson *et al*., 2008); however, the detailed mechanism remains unclear.

In this study, we hypothesize that LR after PH may accelerate the precursor neoplastic lesions to progress to HCC, leading to the *de novo* HCC recurrence. To confirm this hypothesis, an HBx transgenic mouse model was established and PH was performed. Western blotting was carried out to measure the expression profiles of a number of LR‐related SOCS family proteins, growth factors, and transcription factors in the remnant livers at various time points following PH. By comparative analysis of the data between PH and non‐PH groups of mice, our results may provide the potential mechanism for the high *de novo* recurrence of HCC after PH. Intervention in the interaction of LR‐related factors with viral oncoproteins would be considered to improve the prognosis of HCC treatment by PH surgery.

## 2 Material and methods

### 2.1 Transgenic mice and partial hepatectomy

**Figure 1:**
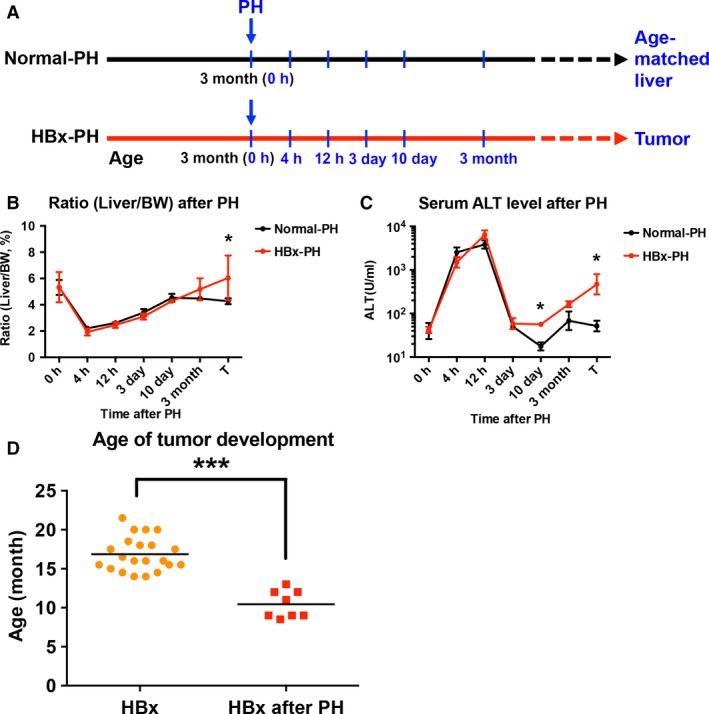
PH‐accelerated tumor development in HBx transgenic mice. (A) Schematic overview of the experimental design of PH in HBx transgenic and non‐transgenic (normal) mice. PH was performed in 3‐month‐old HBx transgenic and non‐transgenic mice. After PH, samples for analyses of liver‐to‐bodyweight (Liver/BW) ratio (B) and serum ALT level (C) were harvested at 0 h, 4 h, 12 h, 3 days, 10 days, and 3 months until tumor formation in HBx transgenic and age‐matched non‐transgenic mice. Three mice were assayed at the time points of 0 h, 4 h, 12 h, 3 days, 10 days, and 3 months, and six mice were assayed when a tumor developed after PH. The various time points after PH are shown in blue. (D) Statistical comparison of the age of tumor formation between PH (*n* = 8) and non‐PH (*n* = 21) groups of HBx transgenic mice showed that PH significantly accelerated tumor formation from the mean age of 16.9 to 10.4 months. Statistically significant difference was indicated (*P < 0.05, ***P < 0.001).

The transgenic mice overexpressing HBx in liver were established by Professor Ting‐Fen Tsai's laboratory (Wu *et al*., 2006). Briefly, the HBx transgenic mice were generated in the C57BL/6 background and the HBx transgene was driven by the liver‐specific albumin promoter. All the animal experiments were performed under the approval of the Institutional Animal Care and Use Committees of the National Cheng Kung University and the National Health Research Institutes. PH was performed according to previously described protocols (Mitchell and Willenbring, 2008). In brief, 3‐month‐old HBx transgenic and non‐transgenic mice were subjected to PH. During PH, the median and left lateral lobes of livers of mice were removed. After PH, mice were followed up and sacrificed to harvest the remnant livers for biochemical and histological analyses at various time points (4 h, 12 h, 3 days, 10 days, 3 months) until tumor formation (Fig. 1A). Upon tumor formation in HBx transgenic mice, age‐matched non‐transgenic mice were also sacrificed for analysis. The time point during PH was set as 0 h and the surgically removed livers were taken to provide information on the initial status of mice and serve as a control after PH. Serum alanine aminotransferase (ALT) level was measured by FUJIFILM DR‐CHEM slides using FUJIFILM DR‐CHEM 3500 machine (FUJIFILM Corporation, Tokyo, Japan).

### 2.2 Western blot analysis

As PH was carried out in 3‐month‐old mice, 3‐month‐old non‐PH mice were selected for age‐matched comparison with the mice at the time points of 0 h, 4 h, 12 h, 3 days, and 10 days after PH, and 6‐month‐old non‐PH mice were selected for age‐matched comparison with the mice at 3M after PH. Because the HBx transgenic mice receiving PH developed tumors at the mean age of 10.4 months (range 8.5–13.0; Fig. 1D), we chose 12‐month‐old non‐PH mice for age‐matched comparison with the mice at the time point of tumor formation following PH. Western blot analysis was performed as previously described (Teng *et al*., 2011). Briefly, total proteins in mice liver tissues were extracted with lysis buffer containing protease and phosphatase inhibitor cocktail (Roche Diagnostics, Mannheim, Germany). Equal amounts of proteins for each sample were resolved on sodium dodecyl sulfate‐polyacrylamide gels and transferred to polyvinylidene difluoride membranes. Membranes were incubated with primary antibodies, followed by secondary antibodies, and then developed by an enhanced chemiluminescence system (Amersham Pharmacia Biotech, Amersham, UK). The primary antibodies used in this study were anti‐SOCS1 (ab83493), anti‐SOCS5 (ab56649), anti‐SOCS6 (ab157168), anti‐Smad2 (ab33875), anti‐p‐Smad2 (Ser467) (ab53100), anti‐Smad3 (ab28379), anti‐HGF (ab83760), anti‐Myc (ab32072; Abcam, Cambridge, UK), anti‐SOCS3 (2932), anti‐STAT3 (9139), anti‐p‐STAT3 (Tyr705) (9145), anti‐p‐STAT3 (Ser727) (9134), anti‐TGF‐β (3711), anti‐p‐Smad3 (Ser423/425) (9520; Cell Signaling Technology, Danvers, MA, USA), anti‐SOCS2 (sc‐9022), anti‐SOCS7 (sc‐5609), anti‐PGC1‐α (SC‐13067), anti‐Smad4 (sc‐7966), anti‐VEGF‐A (sc‐152; Santa Cruz Biotechnology, Santa Cruz, CA, USA), anti‐SOCS4 (MAB5628; R&D Systems, Minneapolis, MN, USA), anti‐β‐Catenin (610153; BD Biosciences, Bedford, MA, USA), and anti‐β‐Actin (MAB1501; Millipore, Billerica, MA, USA). β‐Actin was used as the internal control.

### 2.3 Statistical analysis

The significance of the age of tumor development between PH and non‐PH groups of HBx transgenic mice was determined by unpaired *t*‐test (*P < 0.05, **P < 0.01, ***P < 0.001). The significance of protein expression of each LR‐related factor between HBx transgenic and non‐transgenic mice with or without PH was determined by unpaired *t*‐test. Data represent the mean with standard deviation (SD) error bar. By comparative analysis of the expression data between PH and non‐PH groups of mice, the LR‐related factors whose expression was significantly changed and strictly related to PH in the HBx transgenic mice were identified.

## 3 Data accessibility

All data generated or analyzed during this study are included in this published article and its supplementary information files.

## 4 Results

### 4.1 The incidence and mean age of HCC formation in HBx transgenic mice

To verify the role of HBx in tumorigenesis, we first investigated the incidence and mean age of HCC formation in HBx transgenic mice. As shown in Table 1, the HBx transgenic mice developed HCC with a 100% incidence in livers of both males (*n* = 20) and females (*n* = 18). None of non‐transgenic mice was observed to develop tumor. The tumor development of HBx transgenic mice was faster in males (mean age 16.9 months; range 14.0–21.5 months) than in females (mean age 21.2 months; range 14.0–27.0 months; *P* < 0.05). Therefore, we chose male HBx transgenic mice to perform PH for further experiments. The HBx transgenic mice receiving PH were used as a model to mimic the progression of HCC recurrence after PH.

**Table 1:** The incidence and mean age of HCC formation in HBx transgenic mice

Transgenic mice	Gender	Number of mice studied	Number of mice with tumor	Tumor incidence	Mean age of tumor formation, months
HBx	Male	20	20	100%	16.9
	Female	18	18	100%	21.2

### 4.2 PH‐accelerated tumor development in HBx transgenic mice

To examine the effect of PH on HBx‐mediated tumorigenesis, the liver‐to‐bodyweight ratio as well as serum ALT level of both HBx transgenic and non‐transgenic mice were recorded at various time points until tumor formation after PH. As shown in Fig. 1B, the ratio of liver‐to‐bodyweight showed no significant difference between HBx transgenic and non‐transgenic mice within 3 months after PH but it was significantly increased in HBx transgenic mice compared with non‐transgenic mice upon tumor formation after PH. Serum ALT level was significantly elevated in HBx transgenic mice compared with non‐transgenic mice at 10 days and tumor formation after PH (Fig. 1C). Interestingly, we observed that the mean age of tumor formation in HBx transgenic mice after PH was significantly accelerated from 16.9 months (range 14.0–21.5) in the non‐PH group (*n* = 21) to 10.4 months (range 8.5–13.0) in the PH group (*n* = 8; Fig. 1D). None of non‐transgenic mice developed tumor during the study period after PH. The results of our animal experiments suggest that hepatocarcinogenesis was accelerated in HBx transgenic mice after PH.

### 4.3 Dynamic expression of LR‐related SOCS family proteins in the progression of HBx transgenic tumorigenesis after PH

**Figure 2:**
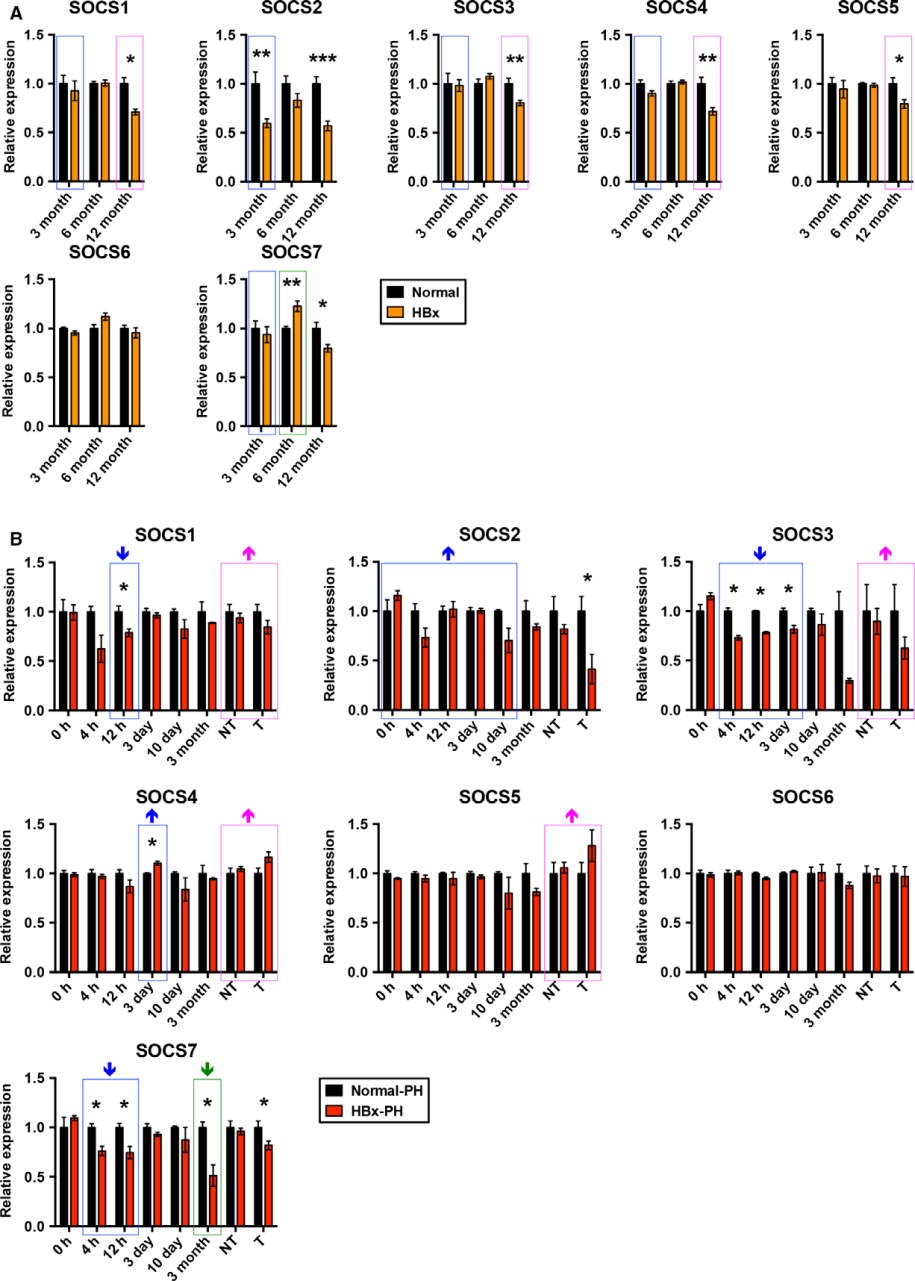
Dynamic expression of LR‐related SOCS family proteins in the progression of HBx transgenic tumorigenesis after PH. The expression of SOCS family proteins in livers of HBx transgenic and non‐transgenic mice with or without PH was examined by western blotting at the indicated time points, followed by quantitative and statistical analysis (*P < 0.05, **P < 0.01, ***P < 0.001). The expression of each SOCS protein in HBx transgenic livers, non‐tumors (NT) or tumors (T) was correlated with that in non‐transgenic (normal) livers. The expression changes of each SOCS protein in HBx transgenic livers compared with non‐transgenic livers between PH and non‐PH groups of mice are highlighted with the colored boxes corresponding to the age‐matched comparison (blue boxes for PH mice at 0 h, 4 h, 12 h, 3 days, 10 days post‐PH and 3‐month‐old non‐PH mice; green boxes for PH mice at 3 months post‐PH and 6‐month‐old non‐PH mice; magenta boxes for PH mice with tumors and 12‐month‐old non‐PH mice). The upward and downward arrows above each box indicate increased and decreased expression changes in the PH group compared with the non‐PH group of mice, respectively. The number of mice assayed in each group at each time point is shown in Figs S1 and S2.

To investigate the molecular basis of the acceleration of HBx transgenic tumorigenesis after PH, the protein expression level of SOCS family members (SOCS1–7) in livers at various time points until tumor formation after PH was examined by western blot analysis in both HBx transgenic and non‐transgenic mice (Fig. S1). For comparative analysis, the expression of SOCS family proteins was also detected in the age‐matched HBx transgenic and non‐transgenic mice without PH (Fig. S1). As shown in Fig. 2, SOCS1 expression was significantly downregulated in HBx transgenic livers compared with non‐transgenic livers at 12 h post‐PH rather than in the age‐matched 3‐month‐old non‐PH transgenic livers. Conversely, although SOCS1 expression was significantly downregulated in 12‐month‐old non‐PH HBx transgenic livers compared with non‐transgenic livers, no significant difference of SOCS1 expression was observed between transgenic and non‐transgenic livers at the time point of tumor formation after PH. Compared with non‐transgenic livers, SOCS2 expression was significantly downregulated in 3‐month‐old non‐PH HBx transgenic livers but not in the transgenic livers at the time points within 10 days after PH. Conversely, SOCS2 expression showed consistent downregulation in HBx transgenic tumors post‐PH as well as in the age‐matched 12‐month‐old non‐PH transgenic livers compared with non‐transgenic livers. The expression of SOCS3 was significantly downregulated in HBx transgenic livers compared with non‐transgenic livers at the relatively early time points of 4 h, 12 h, and 3 days after PH rather than the age‐matched 3‐month‐old non‐PH transgenic livers. Conversely, SOCS3 expression was significantly downregulated in 12‐month‐old non‐PH HBx transgenic livers compared with non‐transgenic livers but not in the transgenic livers at the time point of tumor formation after PH. The expression of SOCS4 was significantly increased in HBx transgenic livers at 3 days post‐PH rather than in the age‐matched 3‐month‐old non‐PH transgenic livers compared with non‐transgenic livers. However, no significant difference in SOCS4 expression was observed between HBx transgenic and non‐transgenic livers at the time point of tumor formation post‐PH, although SOCS4 expression was significantly downregulated in the age‐matched 12‐month‐old non‐PH transgenic livers compared with non‐transgenic livers. SOCS5 expression was significantly downregulated in 12‐month‐old non‐PH HBx transgenic livers compared with non‐transgenic livers but not in the transgenic livers at the time point of tumor formation after PH. No significant difference in SOCS6 expression was observed between HBx transgenic and non‐transgenic livers with or without PH during the study periods. Compared with non‐transgenic livers, SOCS7 expression was significantly downregulated in HBx transgenic livers at the early time points of 4 and 12 h and the late time point of 3 months as well as in transgenic tumors after PH. However, SOCS7 expression was significantly upregulated in 6‐month‐old and downregulated in 12‐month‐old non‐PH transgenic livers compared with non‐transgenic livers.

### 4.4 Dynamic expression of LR‐related growth and transcription factors in the progression of HBx transgenic tumorigenesis after PH

**Figure 3:**
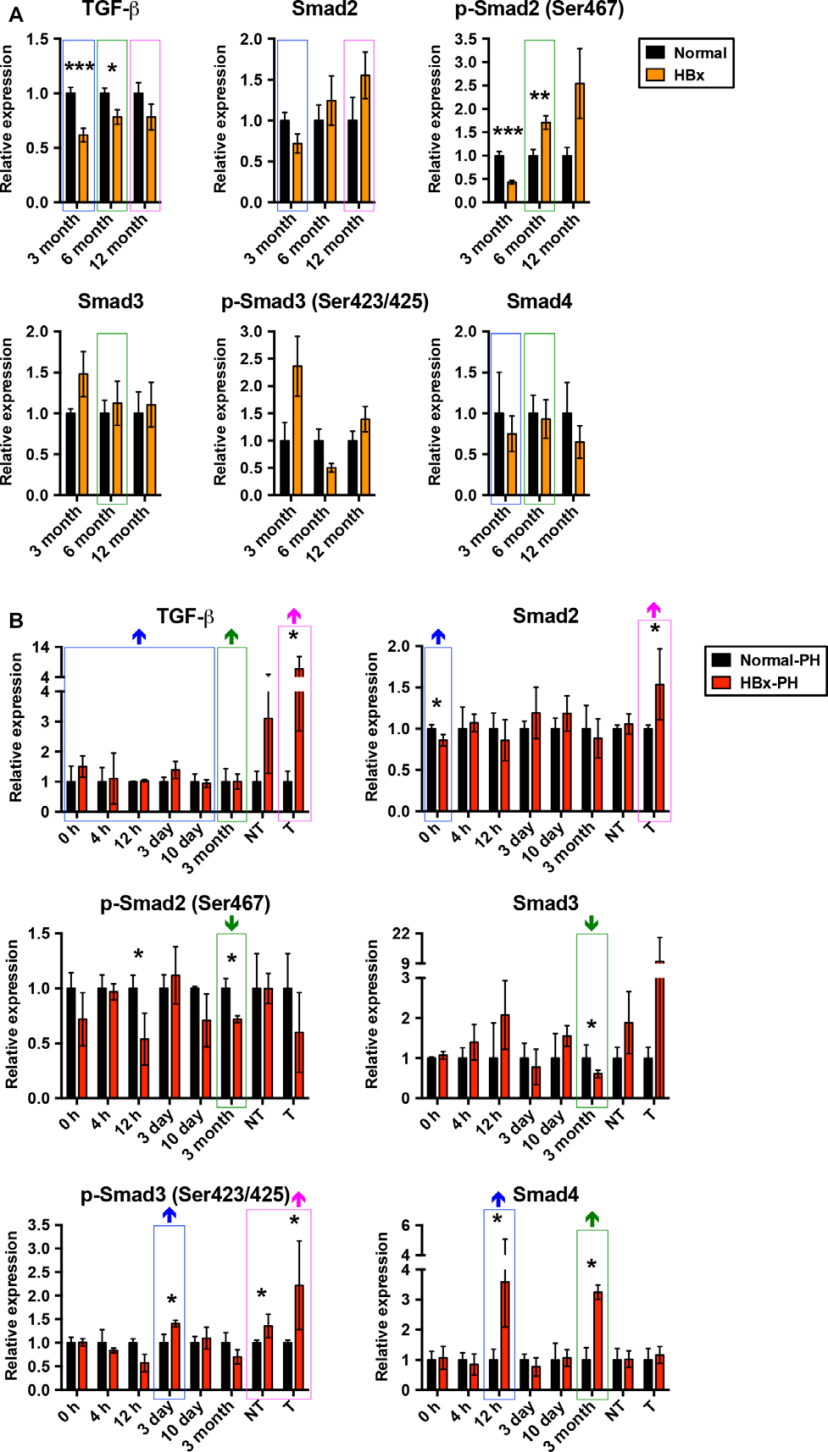
Dynamic expression of LR‐related TGF‐β/Smad pathway in the progression of HBx transgenic tumorigenesis after PH. The expression of TGF‐β and its downstream Smad proteins was examined by western blotting at the indicated time points, followed by quantitative and statistical analysis (*P < 0.05, **P < 0.01, ***P < 0.001). The expression of each signal molecule in HBx transgenic livers, non‐tumors (NT) or tumors (T) correlated with that in non‐transgenic (normal) livers. The expression changes of each signal molecule in HBx transgenic livers compared with non‐transgenic livers between PH and non‐PH groups of mice are highlighted, with the colored boxes corresponding to the age‐matched comparison as shown in Fig. 2. The upward and downward arrows above each box indicate the increased and decreased expression changes in the PH group compared with the non‐PH group of mice, respectively. The number of mice assayed in each group at each time point is shown in Figs S1 and S3.

Next, we examined the protein expression profiles of several growth and transcription factors, including the TGF‐β/Smad pathway, VEGF‐A, HGF, Myc, PGC1‐α, STAT3, and β‐Catenin in livers of HBx transgenic and non‐transgenic mice with or without PH by western blot analysis (Figs S2 and S3). As shown in Fig. 3, compared with non‐transgenic livers, TGF‐β expression was significantly upregulated in transgenic tumors after PH, although its expression was significantly downregulated in 3‐month‐old and 6‐month‐old non‐PH transgenic livers. Smad2 expression was significantly downregulated in HBx transgenic livers at 0 h but upregulated in transgenic tumors after PH compared with the non‐PH transgenic livers. The expression of Ser467‐phosphorylated Smad2 was significantly downregulated in HBx‐transgenic livers rather than in the non‐PH transgenic livers, compared with non‐transgenic livers at 12 h and 3 months post‐PH, although its expression was significantly downregulated and upregulated in the age‐matched 3‐ and 6‐month‐old non‐PH transgenic livers, respectively. Smad3 expression was significantly downregulated in HBx transgenic livers at 3 months post‐PH but not in the non‐PH transgenic livers compared with non‐transgenic livers. The expression of Ser423/425‐phosphorylated Smad3 was significantly upregulated in HBx transgenic livers at 3 days as well as in transgenic non‐tumors and tumors after PH rather than in the non‐PH transgenic livers compared with non‐transgenic livers. Smad4 expression was significantly upregulated in HBx transgenic livers at 12 h and 3 months post‐PH but not in the non‐PH transgenic livers compared with non‐transgenic livers.

**Figure 4:**
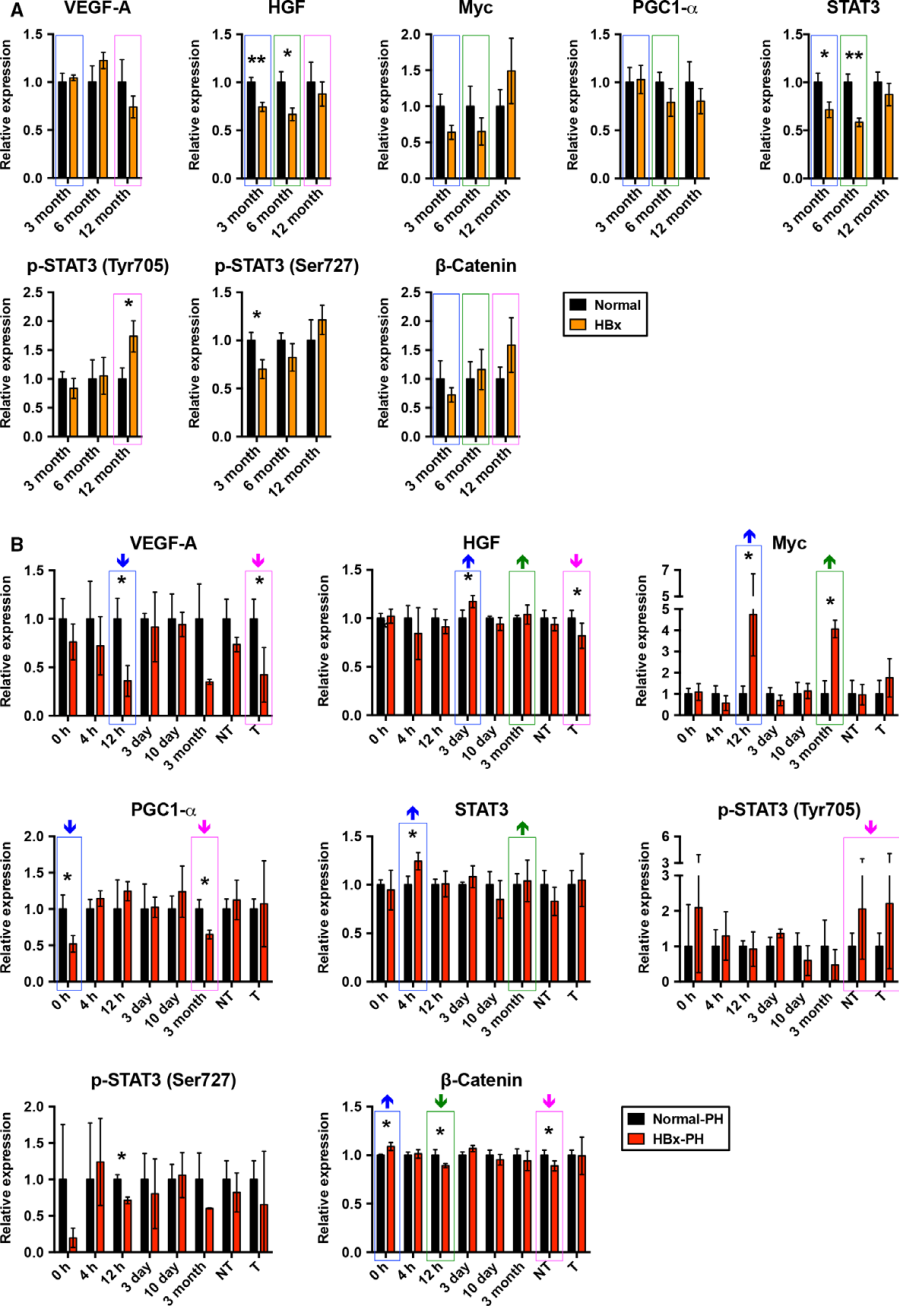
Dynamic expression of LR‐related growth and transcription factors in the progression of HBx transgenic tumorigenesis after PH. The expression of several LR‐related growth and transcription factors was examined by western blotting at the indicated time points, followed by quantitative and statistical analysis (*P < 0.05, **P < 0.01). The expression of each LR‐related factor in HBx transgenic livers, non‐tumors (NT) or tumors (T) correlated with that in non‐transgenic (normal) livers. The expression changes of each LR‐related factor in HBx transgenic livers compared with non‐transgenic livers between the PH and non‐PH groups of mice are highlighted, with the colored boxes corresponding to the age‐matched comparison as shown in Fig. 2. The upward and downward arrows above each box indicate the increased and decreased expression changes in PH group compared with non‐PH group of mice, respectively. The number of mice assayed in each group at each time point is shown in Figs S1 and S3.

As shown in Fig. 4, compared with non‐transgenic livers, VEGF‐A expression was significantly downregulated in HBx transgenic livers at 6 months and in transgenic tumors after PH rather than the age‐matched transgenic livers. HGF expression was significantly upregulated in HBx transgenic livers at 3 days but downregulated in transgenic tumors after PH, although its expression was significantly downregulated in 3‐ and 6‐month‐old non‐PH transgenic livers compared with non‐transgenic livers. Myc expression was significantly upregulated in HBx transgenic livers compared with non‐transgenic livers at 12 h and 3 months post‐PH but not in the age‐matched non‐PH transgenic livers. PGC1‐α expression was significantly downregulated in HBx transgenic livers at 0 h and 3 months post‐PH but not in the age‐matched non‐PH transgenic livers compared with non‐transgenic livers. STAT3 expression was significantly upregulated in HBx transgenic livers at 4 h post‐PH but downregulated in 3‐ and 6‐month‐old non‐PH transgenic livers compared with non‐transgenic livers. The expression of Tyr705‐phosphorylated STAT3 showed no significant difference between HBx transgenic and non‐transgenic livers at any of the examined time points after PH but it was upregulated in 12‐month‐old non‐PH transgenic livers compared with non‐transgenic livers. The expression of Ser727‐phosphorylated STAT3 was significantly downregulated in HBx transgenic livers at 12 h post‐PH as well as in the age‐matched 3‐month‐old non‐PH transgenic livers compared with non‐transgenic livers. Compared with non‐transgenic livers, β‐Catenin expression was significantly upregulated in HBx transgenic livers at 0 h but downregulated in transgenic livers at 12 h and in transgenic non‐tumors after PH. However, no significant difference of β‐Catenin expression was observed between HBx transgenic and non‐transgenic livers in the non‐PH mice.

## 5 Discussion

**Figure 5:**
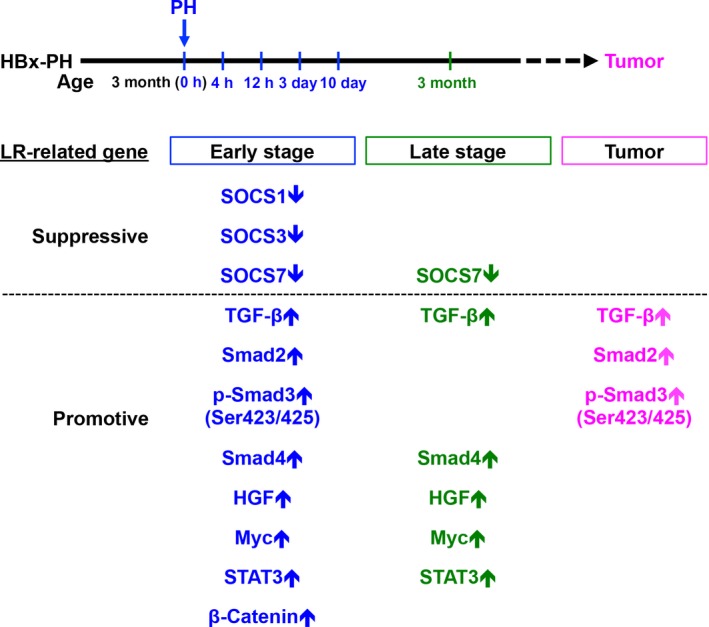
Proposed schematic model for the role of PH‐induced LR in the acceleration of HBx transgenic tumorigenesis. In the HBx transgenic mouse model receiving PH, the expression level of several LR‐related genes, whose protein products exhibit either suppressive or promotive effect on LR, is either downregulated (downward arrows) or upregulated (upward arrows) in the remnant livers from the early time points (0 h, 4 h, 12 h, 3 days, and 10 days; blue‐colored) to the late time point (3 months; green‐colored) until tumor formation (magenta‐colored) after PH in comparison with the non‐PH mice. The combined effect of downregulation of the indicated suppressive molecules and upregulation of the indicated promotive molecules at the indicated stages following PH may induce a LR‐initiated growth promotion on the remnant livers, contributing to the acceleration of HBx transgenic tumorigenesis.

Liver has the remarkable capacity to regenerate after surgical removal or injury. The unique regenerative property of liver constitutes a dilemma in surgical treatment of HCC, considering the potential role of PH‐induced LR in the acceleration of tumorigenesis in the remnant liver, which harbors precancerous lesions such as HBV infection. In this study, we used HBx transgenic mice to mimic the surgical resection of HBV‐related HCC patients. This HBx transgenic mouse model provides a more natural course of tumor development in chronic HBV infection. Previous studies were usually performed on normal rodents either receiving transplanted tumor cells or using chemicals to induce tumor development (de Jong *et al*., 1995; Picardo *et al*., 1998). Using this model, we observed that the mean age of tumor formation in HBx transgenic livers was accelerated from 16.9 to 10.4 months after PH, implying that PH‐induced LR may accelerate the progression of HBx‐induced tumorigenesis, a condition mimicking the observation of *de novo* recurrence of HBV‐related HCC after surgical resection. Our results may provide an explanation for the high *de novo* recurrence of HBV‐related HCC after PH, probably through induction of the sequential changes of LR‐related SOCS family proteins, growth factors, and transcription factors, which promote growth on the precancerous remnant liver (Fig. 5; Chok *et al*., 2011; Iizuka *et al*., 2003; Sonnenblick and Zahavi, 2017).

Activation of LR after PH has been reported to be mediated by some important clusters of networks: cytokines, metabolic signals, and tight regulation of growth factors and its receptors (Kurinna and Barton, 2011). However, the mechanism of LR in *de novo* HCC recurrence after PH is still poorly understood and the prediction biomarkers are not well defined. In this study, the results revealed that the dysregulation of LR‐related SOCS family proteins may play one role. The expression of three SOCS proteins (SOCS1, SOCS3, and SOCS7) was remarkably downregulated in livers of HBx transgenic mice compared with non‐transgenic mice from the early time points (4 h, 12 h, and 3 days) to the late time points (3 months) after PH in comparison with the age‐matched non‐PH mice. This result is consistent with the anti‐tumor role of SOCS family proteins in LR after PH. By acting on cytokines and growth factors that promote hepatocyte proliferation, mice deficient in either SOCS1 or SOCS3 were shown to exhibit an accelerated rate of LR after PH (Gui *et al*., 2011; Riehle *et al*., 2008). In addition, SOCS1 and SOCS3 have been implicated in HCC (Niwa *et al*., 2005; Yoshida *et al*., 2004; Yoshikawa *et al*., 2001). The gene loss and epigenetic silencing of SOCS1 and SOCS3 frequently occurred in HBV‐related HCC patients (Niwa *et al*., 2005; Zhang *et al*., 2014). Moreover, one recent study reported that HBx could downregulate the expression of SOCS1 through enhancing the methylation of SOCS1 gene promoter (Fu *et al*., 2016). The biological function of SOCS7 protein remains to be determined. Considering that SOCS family proteins function as a terminator of LR, the suppression of SOCS1, SOCS3, and SOCS7 expression in remnant livers may be involved in the accelerated tumor development in HBx transgenic mice after PH.

Besides SOCS family proteins, in this study we examined the expression of LR‐related TGF‐β and its downstream Smad proteins. The TGF‐β/Smad pathway plays essential roles in regulation of a wide variety of cellular functions, including proliferation, differentiation, migration, and apoptosis (Drabsch and ten Dijke, 2012). Several studies have suggested that dysregulation of this pathway contributes to all stages of liver disease progression, from initial liver injury through inflammation and fibrosis to cirrhosis and HCC (Fabregat *et al*., 2016; Giannelli *et al*., 2014; Meindl‐Beinker *et al*., 2012). Consistent with these findings, our results showed that the expression of TGF‐β, Smad2, Ser423/425‐phosphorylated Smad3, and Smad4 was remarkably increased in livers of HBx transgenic mice compared with non‐transgenic mice from the early time points (4 h, 12 h, 3 days, and 10 days) to the late time points (3 months) even up to tumor formation after PH in comparison with the age‐matched non‐PH mice. The sustained activation of the TGF‐β/Smad pathway during the entire period following PH implies that HBx may affect the TGF‐β/Smad pathway, leading to tumor progression.

In this study, several LR‐related growth and transcription factors were also investigated. HGF, Myc, and β‐Catenin are important for efficient LR through driving cell cycle progression and cell proliferation after PH (Fausto *et al*., 2006; Kurinna and Barton, 2011; Monga *et al*., 2001; Sanders *et al*., 2012). Consistently, our results revealed that the expression of HGF, Myc, and β‐Catenin was significantly upregulated in HBx transgenic livers compared with non‐transgenic mice at both the early (0 h, 12 h, and 3 days) and late (3 months) time points after PH in comparison with the age‐matched non‐PH mice, implying that HBx may enhance the progression of HCC after PH by positively regulating these signal molecules. STAT3 is rapidly induced in livers in the first few hours after PH and is required for the transcriptional activation of many immediate/early growth response genes (Cressman *et al*., 1995; Moh *et al*., 2007). In addition, STAT3 has been reported to act as an oncogenic factor to promote tumor growth in liver tumorigenesis (Wang *et al*., 2011). Consistent with these reports, our data also showed that the expression of STAT3 was significantly upregulated in HBx transgenic livers compared with non‐transgenic livers at both the early (4 h) and late (3 months) time points after PH in comparison with the age‐matched non‐PH mice, suggesting that STAT3 may function as a transcriptional activator at the early stage and an oncogenic factor at the late stage of HBx transgenic tumorigenesis after PH.

## 6 Conclusions

In this study we demonstrated that use of PH as a curative therapeutic treatment in the HBx transgenic mice could accelerate tumor development, an outcome which is similarly observed in clinical practice. Until now, PH has been a potentially curative procedure for the treatment of HCC. Although diagnostic methods and therapeutic strategies for HCC have been improved in the past decade, the survival and postsurgical recurrence rates are still high. The HBV‐infected hepatocyte seems to become a pre‐existing precancerous lesion that can disrupt the critical regulators (such as SOCSs, TGF‐β/Smad, HGF, Myc, STAT3, and β‐Catenin) in both physiological and tumorigenic processes. The underlying mechanisms by which PH activates hepatocyte pre‐existing precancerous lesions and leads to the enhanced HBx tumorigenesis still need further investigation. Based on our results, the acceleration of the recurrence of HBV‐infected HCC after PH should be carefully verified, and markers for HCC recurrence could be examined in post‐PH surgical specimens to predict the recurrence, which also provides a benefit for HCC patients from the use of neoadjuvant or adjuvant therapy for HBV‐infected HCC after PH.

## Author contributions

CF performed the experiments, analyzed the data, and was a major contributor in writing the manuscript. HY assisted in analyzing the data and writing the manuscript. WC assisted in performing the experiments and analyzing the data. HW assisted in analyzing the data. YH assisted in performing the experiments. IJ and YJ designed the study and composed the manuscript.

## Supporting information


**Fig. S1.** Western blotting of LR‐related SOCS family proteins in livers of HBx transgenic and non‐transgenic mice with or without PH.

**Fig. S2.** Western blotting of LR‐related TGF‐β/Smad pathway in livers of HBx transgenic and non‐transgenic mice with or without PH.

**Fig. S3.** Western blotting of LR‐related growth and transcription factors in HBx transgenic and non‐transgenic mice with or without PH.

## References

[mol212318-bib-0001] Arbuthnot P , Capovilla A and Kew M (2000) Putative role of hepatitis B virus X protein in hepatocarcinogenesis: effects on apoptosis, DNA repair, mitogen‐activated protein kinase and JAK/STAT pathways. J Gastroenterol Hepatol 15, 357–368. 10824878 10.1046/j.1440-1746.2000.02069.x

[mol212318-bib-0002] Bockhorn M , Goralski M , Prokofiev D , Dammann P , Grunewald P , Trippler M , Biglarnia A , Kamler M , Niehues EM , Frilling A *et al* (2007) VEGF is important for early liver regeneration after partial hepatectomy. J Surg Res 138, 291–299. 17275844 10.1016/j.jss.2006.07.027

[mol212318-bib-0003] Bruix J , Sherman M and Practice Guidelines Committee, American Association for the Study of Liver Diseases (2005) Management of hepatocellular carcinoma. Hepatology 42, 1208–1236. 16250051 10.1002/hep.20933

[mol212318-bib-0004] Chisari FV , Klopchin K , Moriyama T , Pasquinelli C , Dunsford HA , Sell S , Pinkert CA , Brinster RL and Palmiter RD (1989) Molecular pathogenesis of hepatocellular carcinoma in hepatitis B virus transgenic mice. Cell 59, 1145–1156. 2598264 10.1016/0092-8674(89)90770-8

[mol212318-bib-0005] Chok KS , Chan SC , Cheung TT , Chan AC , Fan ST and Lo CM (2011) Late recurrence of hepatocellular carcinoma after liver transplantation. World J Surg 35, 2058–2062. 21597889 10.1007/s00268-011-1146-z PMC3152711

[mol212318-bib-0006] Cressman DE , Diamond RH and Taub R (1995) Rapid activation of the Stat3 transcription complex in liver regeneration. Hepatology 21, 1443–1449. 7737651

[mol212318-bib-0007] Drabsch Y and ten Dijke P (2012) TGF‐beta signalling and its role in cancer progression and metastasis. Cancer Metastasis Rev 31, 553–568. 22714591 10.1007/s10555-012-9375-7

[mol212318-bib-0008] El‐Serag HB (2012) Epidemiology of viral hepatitis and hepatocellular carcinoma. Gastroenterology 142, 1264–1273 e1261. 22537432 10.1053/j.gastro.2011.12.061 PMC3338949

[mol212318-bib-0009] Fabregat I , Moreno‐Caceres J , Sanchez A , Dooley S , Dewidar B , Giannelli G , Ten Dijke P and IT‐LIVER Consortium (2016) TGF‐β signalling and liver disease. FEBS J 283, 2219–2232. 26807763 10.1111/febs.13665

[mol212318-bib-0010] Fausto N , Campbell JS and Riehle KJ (2006) Liver regeneration. Hepatology 43, S45–S53. 16447274 10.1002/hep.20969

[mol212318-bib-0011] Fu X , Song X , Li Y , Tan D and Liu G (2016) Hepatitis B virus X protein upregulates DNA methyltransferase 3A/3B and enhances SOCS‐1CpG island methylation. Mol Med Rep 13, 301–308. 26573490 10.3892/mmr.2015.4545

[mol212318-bib-0012] Giannelli G , Rani B , Dituri F , Cao Y and Palasciano G (2014) Moving towards personalised therapy in patients with hepatocellular carcinoma: the role of the microenvironment. Gut 63, 1668–1676. 25053718 10.1136/gutjnl-2014-307323

[mol212318-bib-0013] Grazi GL , Ercolani G , Pierangeli F , Del Gaudio M , Cescon M , Cavallari A and Mazziotti A (2001) Improved results of liver resection for hepatocellular carcinoma on cirrhosis give the procedure added value. Ann Surg 234, 71–78. 11420485 10.1097/00000658-200107000-00011 PMC1421950

[mol212318-bib-0014] Gui Y , Yeganeh M , Ramanathan S , Leblanc C , Pomerleau V , Ferbeyre G , Saucier C and Ilangumaran S (2011) SOCS1 controls liver regeneration by regulating HGF signaling in hepatocytes. J Hepatol 55, 1300–1308. 21703184 10.1016/j.jhep.2011.03.027

[mol212318-bib-0015] Hodgson AJ , Keasler VV and Slagle BL (2008) Premature cell cycle entry induced by hepatitis B virus regulatory HBx protein during compensatory liver regeneration. Cancer Res 68, 10341–10348. 19074903 10.1158/0008-5472.CAN-08-2695 PMC2730779

[mol212318-bib-0016] Hoshida Y , Toffanin S , Lachenmayer A , Villanueva A , Minguez B and Llovet JM (2010) Molecular classification and novel targets in hepatocellular carcinoma: recent advancements. Semin Liver Dis 30, 35–51. 20175032 10.1055/s-0030-1247131 PMC3668687

[mol212318-bib-0017] Iizuka N , Oka M , Yamada‐Okabe H , Nishida M , Maeda Y , Mori N , Takao T , Tamesa T , Tangoku A , Tabuchi H *et al* (2003) Oligonucleotide microarray for prediction of early intrahepatic recurrence of hepatocellular carcinoma after curative resection. Lancet 361, 923–929. 12648972 10.1016/S0140-6736(03)12775-4

[mol212318-bib-0018] de Jong KP , Lont HE , Bijma AM , Brouwers MA , de Vries EG , van Veen ML , Marquet RL , Slooff MJ and Terpstra OT (1995) The effect of partial hepatectomy on tumor growth in rats: in vivo and in vitro studies. Hepatology 22, 1263–1272. 7557880

[mol212318-bib-0019] Kim BY , Choi DW , Woo SR , Park ER , Lee JG , Kim SH , Koo I , Park SH , Han CJ , Kim SB *et al* (2015) Recurrence‐associated pathways in hepatitis B virus‐positive hepatocellular carcinoma. BMC Genom 16, 279. 10.1186/s12864-015-1472-x PMC4448317 25888140

[mol212318-bib-0020] Kishi Y , Hasegawa K , Sugawara Y and Kokudo N (2011) Hepatocellular carcinoma: current management and future development‐improved outcomes with surgical resection. Int J Hepatol 2011, 728103. 21994868 10.4061/2011/728103 PMC3170840

[mol212318-bib-0021] Kurinna S and Barton MC (2011) Cascades of transcription regulation during liver regeneration. Int J Biochem Cell Biol 43, 189–197. 20307684 10.1016/j.biocel.2010.03.013 PMC2923255

[mol212318-bib-0022] Meindl‐Beinker NM , Matsuzaki K and Dooley S (2012) TGF‐beta signaling in onset and progression of hepatocellular carcinoma. Dig Dis 30, 514–523. 23108308 10.1159/000341704

[mol212318-bib-0023] Michalopoulos GK (2010) Liver regeneration after partial hepatectomy: critical analysis of mechanistic dilemmas. Am J Pathol 176, 2–13. 20019184 10.2353/ajpath.2010.090675 PMC2797862

[mol212318-bib-0024] Mitchell C and Willenbring H (2008) A reproducible and well‐tolerated method for 2/3 partial hepatectomy in mice. Nat Protoc 3, 1167–1170. 18600221 10.1038/nprot.2008.80

[mol212318-bib-0025] Moh A , Iwamoto Y , Chai GX , Zhang SS , Kano A , Yang DD , Zhang W , Wang J , Jacoby JJ , Gao B *et al* (2007) Role of STAT3 in liver regeneration: survival, DNA synthesis, inflammatory reaction and liver mass recovery. Lab Invest 87, 1018–1028. 17660847 10.1038/labinvest.3700630

[mol212318-bib-0026] Monga SP , Pediaditakis P , Mule K , Stolz DB and Michalopoulos GK (2001) Changes in WNT/beta‐catenin pathway during regulated growth in rat liver regeneration. Hepatology 33, 1098–1109. 11343237 10.1053/jhep.2001.23786 PMC1821078

[mol212318-bib-0027] Nakashima Y , Nakashima O , Tanaka M , Okuda K , Nakashima M and Kojiro M (2003) Portal vein invasion and intrahepatic micrometastasis in small hepatocellular carcinoma by gross type. Hepatol Res 26, 142–147. 12809942 10.1016/s1386-6346(03)00007-x

[mol212318-bib-0028] Newell P , Villanueva A , Friedman SL , Koike K and Llovet JM (2008) Experimental models of hepatocellular carcinoma. J Hepatol 48, 858–879. 18314222 10.1016/j.jhep.2008.01.008 PMC2990959

[mol212318-bib-0029] Niwa Y , Kanda H , Shikauchi Y , Saiura A , Matsubara K , Kitagawa T , Yamamoto J , Kubo T and Yoshikawa H (2005) Methylation silencing of SOCS‐3 promotes cell growth and migration by enhancing JAK/STAT and FAK signalings in human hepatocellular carcinoma. Oncogene 24, 6406–6417. 16007195 10.1038/sj.onc.1208788

[mol212318-bib-0030] Picardo A , Karpoff HM , Ng B , Lee J , Brennan MF and Fong Y (1998) Partial hepatectomy accelerates local tumor growth: potential roles of local cytokine activation. Surgery 124, 57–64. 9663252

[mol212318-bib-0031] Riehle KJ , Campbell JS , McMahan RS , Johnson MM , Beyer RP , Bammler TK and Fausto N (2008) Regulation of liver regeneration and hepatocarcinogenesis by suppressor of cytokine signaling 3. J Exp Med 205, 91–103. 18158318 10.1084/jem.20070820 PMC2234364

[mol212318-bib-0032] Sanders JA , Schorl C , Patel A , Sedivy JM and Gruppuso PA (2012) Postnatal liver growth and regeneration are independent of c‐myc in a mouse model of conditional hepatic c‐myc deletion. BMC Physiol 12, 1. 22397685 10.1186/1472-6793-12-1 PMC3353165

[mol212318-bib-0033] Sonnenblick A and Zahavi T (2017) Accelerated carcinogenesis following liver resection in chronically inflamed livers: a window of opportunity for treatment. Biomed Rep 6, 545–548. 28515912 10.3892/br.2017.882 PMC5431396

[mol212318-bib-0034] Tang ZY , Ye SL , Liu YK , Qin LX , Sun HC , Ye QH , Wang L , Zhou J , Qiu SJ , Li Y *et al* (2004) A decade's studies on metastasis of hepatocellular carcinoma. J Cancer Res Clin Oncol 130, 187–196. 14685850 10.1007/s00432-003-0511-1 PMC12161827

[mol212318-bib-0035] Teng CF , Hsieh WC , Yang CW , Su HM , Tsai TF , Sung WC , Huang W and Su IJ (2016) A biphasic response pattern of lipid metabolomics in the stage progression of hepatitis B virus X tumorigenesis. Mol Carcinog 55, 105–114. 25594851 10.1002/mc.22266

[mol212318-bib-0036] Teng CF , Wu HC , Tsai HW , Shiah HS , Huang W and Su IJ (2011) Novel feedback inhibition of surface antigen synthesis by mammalian target of rapamycin (mTOR) signal and its implication for hepatitis B virus tumorigenesis and therapy. Hepatology 54, 1199–1207. 21735472 10.1002/hep.24529

[mol212318-bib-0037] Wang H , Lafdil F , Wang L , Park O , Yin S , Niu J , Miller AM , Sun Z and Gao B (2011) Hepatoprotective versus oncogenic functions of STAT3 in liver tumorigenesis. Am J Pathol 179, 714–724. 21684247 10.1016/j.ajpath.2011.05.005 PMC3157203

[mol212318-bib-0038] Wu BK , Li CC , Chen HJ , Chang JL , Jeng KS , Chou CK , Hsu MT and Tsai TF (2006) Blocking of G1/S transition and cell death in the regenerating liver of Hepatitis B virus X protein transgenic mice. Biochem Biophys Res Commun 340, 916–928. 16403455 10.1016/j.bbrc.2005.12.089

[mol212318-bib-0039] Wu HC , Tsai HW , Teng CF , Hsieh WC , Lin YJ , Wang LH , Yuan Q and Su IJ (2014) Ground‐glass hepatocytes co‐expressing hepatitis B virus X protein and surface antigens exhibit enhanced oncogenic effects and tumorigenesis. Hum Pathol 45, 1294–1301. 24767856 10.1016/j.humpath.2013.10.039

[mol212318-bib-0040] Yen TT , Yang A , Chiu WT , Li TN , Wang LH , Wu YH , Wang HC , Chen L , Wang WC , Huang W *et al* (2016) Hepatitis B virus PreS2‐mutant large surface antigen activates store‐operated calcium entry and promotes chromosome instability. Oncotarget 7, 23346–23360. 26992221 10.18632/oncotarget.8109 PMC5029631

[mol212318-bib-0041] Yin C , Evason KJ , Asahina K and Stainier DY (2013) Hepatic stellate cells in liver development, regeneration, and cancer. J Clin Invest 123, 1902–1910. 23635788 10.1172/JCI66369 PMC3635734

[mol212318-bib-0042] Yoshida T , Ogata H , Kamio M , Joo A , Shiraishi H , Tokunaga Y , Sata M , Nagai H and Yoshimura A (2004) SOCS1 is a suppressor of liver fibrosis and hepatitis‐induced carcinogenesis. J Exp Med 199, 1701–1707. 15197228 10.1084/jem.20031675 PMC2212816

[mol212318-bib-0043] Yoshikawa H , Matsubara K , Qian GS , Jackson P , Groopman JD , Manning JE , Harris CC and Herman JG (2001) SOCS‐1, a negative regulator of the JAK/STAT pathway, is silenced by methylation in human hepatocellular carcinoma and shows growth‐suppression activity. Nat Genet 28, 29–35. 11326271 10.1038/ng0501-29

[mol212318-bib-0044] Zhang X , Wang J , Cheng J , Ding S , Li M , Sun S , Zhang L , Liu S , Chen X , Zhuang H *et al* (2014) An integrated analysis of SOCS1 down‐regulation in HBV infection‐related hepatocellular carcinoma. J Viral Hepat 21, 264–271. 23941364 10.1111/jvh.12137 PMC4229024

[mol212318-bib-0045] Zhong Z , Tsukada S , Rehman H , Parsons CJ , Theruvath TP , Rippe RA , Brenner DA and Lemasters JJ (2010) Inhibition of transforming growth factor‐beta/Smad signaling improves regeneration of small‐for‐size rat liver grafts. Liver Transpl 16, 181–190. 20104486 10.1002/lt.21966 PMC2834418

