# Supplementary figures and images for: Liver regeneration accelerates hepatitis B virus‐related tumorigenesis of hepatocellular carcinoma
Source: Mol Oncol. 2018 May 29;12(7):1175–87. doi: 10.1002/1878-0261.12318 (PMC6026873; doi:10.1002/1878-0261.12318)

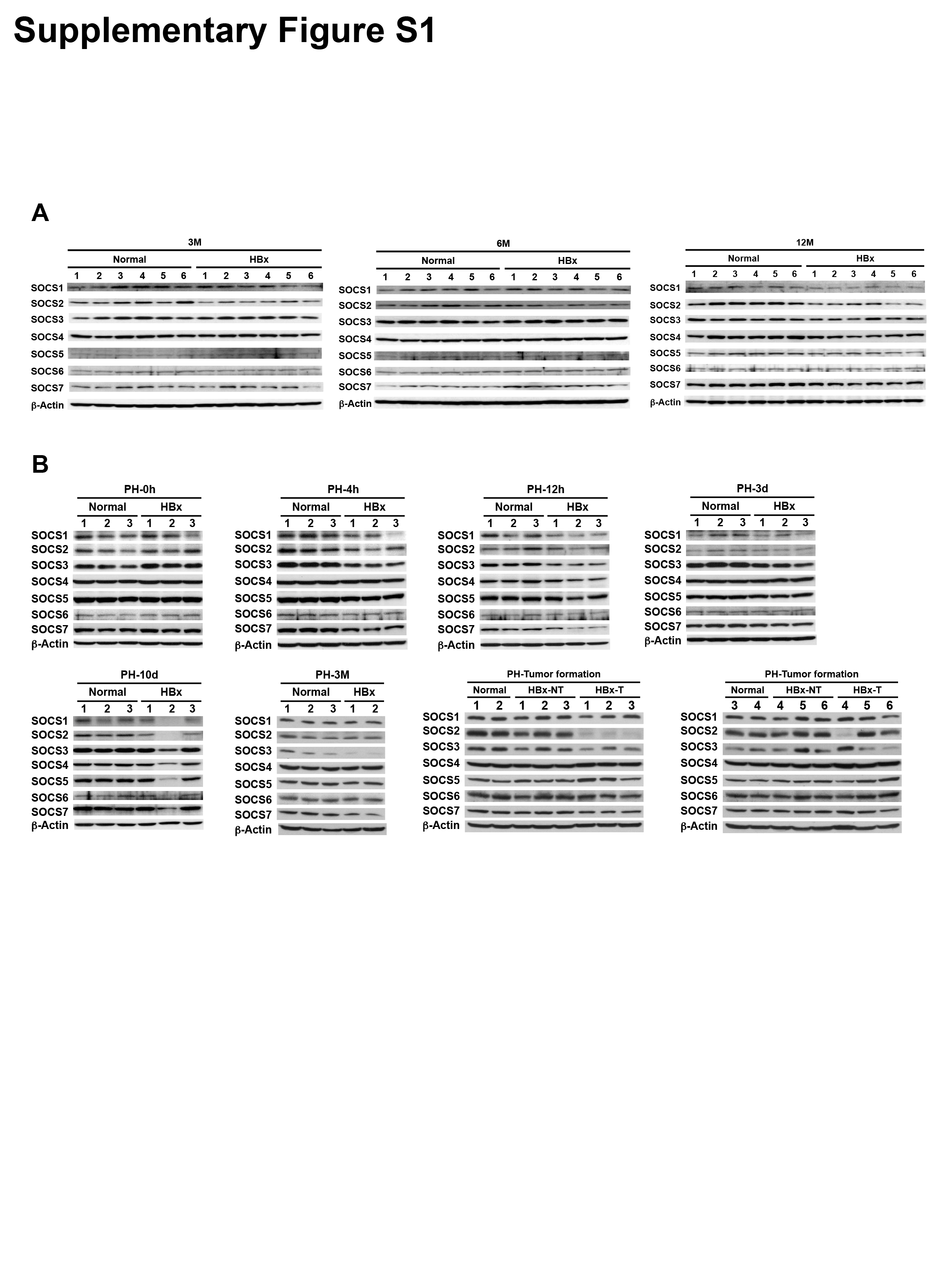

Supplement: Supplementary file 1 — Fig. S1. Western blotting of LR‐related SOCS family proteins in livers of HBx transgenic and non‐transgenic mice with or without PH. [file MOL2-12-1175-s001.tif]

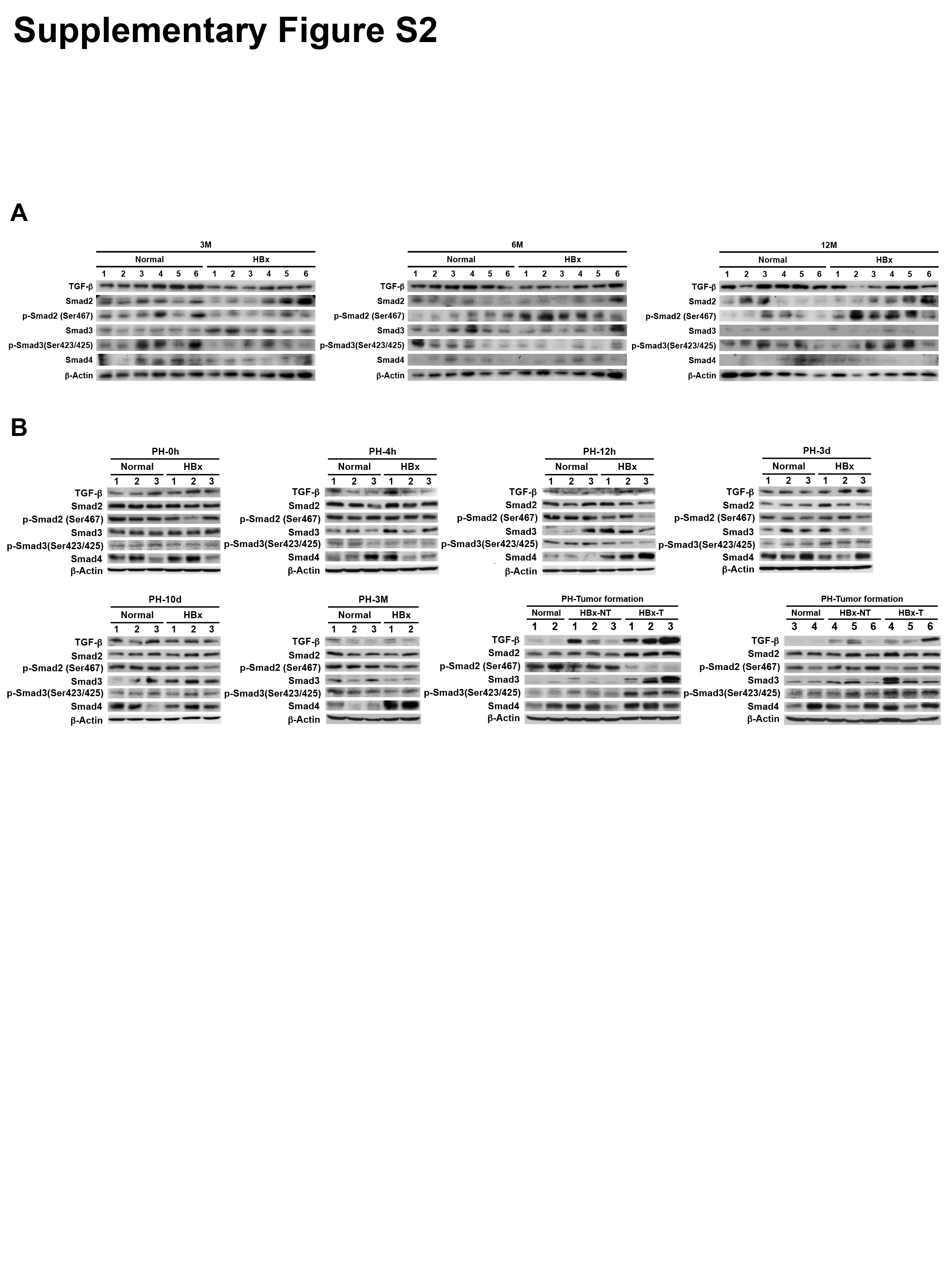

Supplement: Supplementary file 2 — Fig. S2. Western blotting of LR‐related TGF‐β/Smad pathway in livers of HBx transgenic and non‐transgenic mice with or without PH. [file MOL2-12-1175-s002.tif]

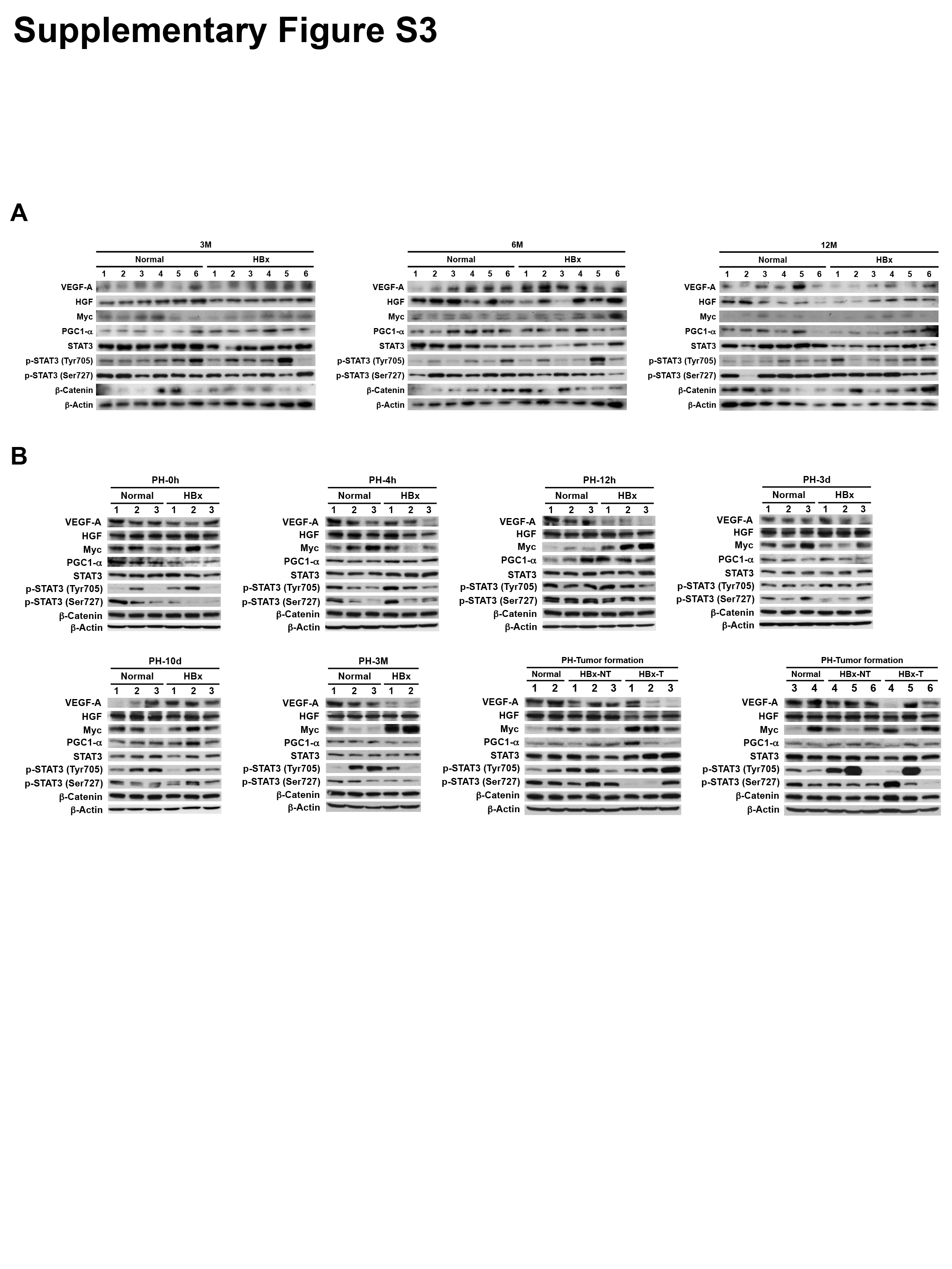

Supplement: Supplementary file 3 — Fig. S3. Western blotting of LR‐related growth and transcription factors in HBx transgenic and non‐transgenic mice with or without PH. [file MOL2-12-1175-s003.tif]
