# Supplementary material for: Liver regeneration accelerates hepatitis B virus‐related tumorigenesis of hepatocellular carcinoma
Source: Mol Oncol. 2018 May 29;12(7):1175–87. doi: 10.1002/1878-0261.12318 (PMC6026873; doi:10.1002/1878-0261.12318)
Supplement: Supplementary file 4 [file MOL2-12-1175-s004.docx]

**Supplementary Figure Legends**

**Supplementary Figure S1. Western blotting of LR-related SOCS family proteins in livers of HBx transgenic and non-transgenic mice with or without PH.** (A) For the age-matched comparison with mice receiving PH, the expression of the SOCS family proteins in livers of 3M-, 6M-, and 12M-old HBx transgenic and non-transgenic (normal) mice without PH was examined by Western blot analysis. At each age, six HBx transgenic and non-transgenic mice were used. (B) At indicated time points after PH, the expression of the SOCS family proteins in livers of HBx transgenic and non-transgenic mice was examined by Western blot analysis. At each time point, three HBx transgenic and non-transgenic mice were used, except two HBx transgenic mice were used at 3M and six HBx transgenic and four non-transgenic mice were used when tumor formation. The PH-0h, 4h, 12h and 3d, 10d, 3m and tumor formation panels in figures S1B, S2B and S3B were derived from the same lysates; thus, the same b-actin loading control is shown across the panels in these three figures. Abbreviations: h, hour; d, day; M, month; NT, non-tumor; T, tumor.

**Supplementary Figure S2. Western blotting of LR-related** **TGF-β/Smad pathway in livers of HBx transgenic and non-transgenic mice with or without PH.** (A) For the age-matched comparison with mice receiving PH, the protein expression of the TGF-β/Smad pathway molecules in livers of 3M-, 6M-, and 12M-old HBx transgenic and non-transgenic (normal) mice without PH was examined by Western blot analysis. At each age, six HBx transgenic and non-transgenic mice were used. (B) At indicated time points after PH, the protein expression of the TGF-β/Smad pathway molecules in livers of HBx transgenic and non-transgenic mice was examined by Western blot analysis. At each time point, three HBx transgenic and non-transgenic mice were used, except two HBx transgenic mice were used at 3M and six HBx transgenic and four non-transgenic mice were used when tumor formation. The PH-0h, 4h, 12h and 3d, 10d, 3m and tumor formation panels in figures S1B, S2B and S3B were derived from the same lysates; thus, the same b-actin loading control is shown across the panels in these three figures. Abbreviations: h, hour; d, day; M, month; NT, non-tumor; T, tumor.

**Supplementary Figure S3. Western blotting of LR-related growth and transcription factors in HBx transgenic and non-transgenic mice with or without PH.** (A) For the age-matched comparison with mice receiving PH, the protein expression of the selected growth and transcription factors in livers of 3M-, 6M-, and 12M-old HBx transgenic and non-transgenic (normal) mice without PH was examined by Western blot analysis. At each age, six HBx transgenic and non-transgenic mice were used. (B) At indicated time points after PH, the protein expression of the selected growth and transcription factors in livers of HBx transgenic and non-transgenic mice was examined by Western blot analysis. At each time point, three HBx transgenic and non-transgenic mice were used, except two HBx transgenic mice were used at 3M and six HBx transgenic and four non-transgenic mice were used when tumor formation. The PH-0h, 4h, 12h and 3d, 10d, 3m and tumor formation panels in figures S1B, S2B and S3B were derived from the same lysates; thus, the same b-actin loading control is shown across the panels in these three figures. Abbreviations: h, hour; d, day; M, month; NT, non-tumor; T, tumor.
